# Modeling Child–Nature Interaction in a Nature Preschool: A Proof of Concept

**DOI:** 10.3389/fpsyg.2018.00835

**Published:** 2018-05-29

**Authors:** Peter H. Kahn, Thea Weiss, Kit Harrington

**Affiliations:** ^1^Department of Psychology, School of Environmental and Forest Sciences, University of Washington, Seattle, WA, United States; ^2^Department of Psychology, University of Washington, Seattle, WA, United States; ^3^Fiddleheads Forest School, University of Washington Botanic Gardens, Seattle, WA, United States

**Keywords:** nature preschools, interaction, interaction patterns, modeling, wild nature, proof of concept, nature language, environmental education

## Abstract

This article provides a proof of concept for an approach to modeling child–nature interaction based on the idea of *interaction patterns*: characterizations of essential features of interaction between humans and nature, specified abstractly enough such that countless different instantiations of each one can occur – in more domestic or wild forms – given different types of nature, people, and purposes. The model draws from constructivist psychology, ecological psychology, and evolutionary psychology, and is grounded in observational data collected through a time-sampling methodology at a nature preschool. Through using a *nature language* that emphasizes ontogenetic and phylogenetic significance, seven keystone interaction patterns are described for this nature preschool: *using one’s body vigorously in nature, striking wood on wood, constructing shelter, being in solitude in nature, lying on earth, cohabiting with a wild animal*, and *being outside in weather*. These 7 interactions patterns are then brought together with 13 other patterns published elsewhere to provide a total of 20 keystone interaction patterns that begin to fill out the model, and to show its promise. Discussion focuses on what the model aims to be in terms of both product and process, on what work the model can currently do, and how to further develop the model.

## Introduction

Nature preschools and forest kindergartens have been increasing in number: from around 25 programs in 2012 to more than 250 in 2017 in the United States alone (North American Association for Environmental Education [NAAEE], 2017). Thus questions in research communities have emerged about these programs, such as how they compare to indoor classrooms on traditional metrics of language development, physical development, executive function, and academic preparation for K-5 schooling. While these questions are important, in our view a complementary line of research is also needed, one that is perhaps even more foundational: to characterize what exactly goes on in nature schools, especially in terms of how children interact with nature. After all, nature is the central environmental feature of nature schools.

To date, many such characterizations have focused on different forms of children’s play in nature (Ginsburg, 2007; Brown and Kaye, 2017; Morrissey et al., 2017). For example, Sobel (2008) postulated the existence of seven universal play motifs: going on adventures, descending into fantasies, shaping small worlds, developing friendships with animals, following paths and figuring out shortcuts, making forts and special places, and playing hunting and gathering games. This is valuable work, and it follows a growing awareness, with roots to Vygotsky (1978), that children’s play in nature is not only diverse, but that it provides the mechanism for many important developmental outcomes.

That said, we believe there is much to be gained by expanding our understanding of children’s engagement with the natural world beyond the scope of play. After all, there are many types of interactions, such as when a child splashes water on her face from a creek to cool down on a hot summer’s day, or retreats to a solitary spot in nature and sits under a tree to regain composure after a conflict, which do not seem well characterized in terms of a play motif.

Thus in this article we bring forward an approach to modeling child–nature interaction – based on the idea of *interaction patterns*. We first provide a psychological basis for our model, drawing on psychological constructivism with roots to Jean Piaget, ecological psychology with roots to James Gibson, and evolutionary psychology with roots to E. O. Wilson. Next we discuss what interaction patterns are, how they can be enacted along a continuum from wild to domestic, and the idea of keystone interaction patterns. From there we specify the form of scientific model that we are proposing. Then we move into the empirical part of this article as we seek to provide a proof of concept for modeling child–nature interaction. We do so by analyzing observational data that we have collected in a nature-based preschool, and provide an account (what we call a nature language) of 7 keystone interaction patterns at this preschool. We then synthesize our keystone interaction patterns with 13 other such patterns (recently published elsewhere) to show that child–nature interaction can be successfully modeled in this way, leading to characterizations with prima facie validity and testable hypotheses for future experimental research.

To be clear, in this current research we do not test a hypothesis. Rather, we offer a proof of concept based on qualitative analysis of original empirical observational data. Thus stylistically we do not have the traditional “methods/results” sections in this article, but sections that we believe provide an effective exposition of our proof of concept.

## The Psychological Grounding for Interaction Patterns

Our model is based on the idea of interaction patterns. Before discussing interaction patterns, it is useful to delineate briefly the psychological grounding for them, which is based on constructivist psychology, ecological psychology, and evolutionary psychology.

### Constructivist Psychology

A large body of evidence shows that the child’s developing conceptual knowledge of the physical and logical world is neither simply a product of innate biological programming (endogenous theories) nor simply a product of cultural learning (exogenous theories); rather it requires the child constructing the knowledge for herself through repeated interactions with the physical and social entities of the world (Piaget, 1952/1963; Langer, 1969; Piaget, 1983; Turiel, 1983).

According to Piaget (1983), the mechanism for the construction of knowledge involves the coordination of two complementary cognitive processes: of assimilation and accommodation. Assimilation is the process that seeks to fit new information into existing cognitive schemas, perceptions, and understandings, while accommodation is the process that adjusts, reorients, and revises those schemas, perceptions, and understandings to account for aspects of the new information that is not readily assimilated. This process is motivated by what is called the mechanism of disequilibration (Kohlberg, 1969, 1971; Piaget, 1983). In other words, through interaction with the environment the child comes to recognize – in daily minor and sometimes major ways – that her current understandings of the world are not able to take into account her previous understandings, and she becomes unsettled. The disequilibrated state is not a comfortable state. Thus the child seeks to construct a more adequate and conceptually sophisticated understanding that solves the problems at hand.

### Ecological Psychology

Along complementary lines, in the theory of ecological psychology, Gibson (1979/1986) postulated that the world is perceived by the individual not only in terms of shapes, spatial relationships, and logical properties, but also in terms of possibilities for action. Gibson proposed that it is our direct perception of information specifying the environment in relation to ourselves – the *affordances* of the environment – that guides our understanding of our surroundings. Affordances are dependent on a reciprocal relationship between the environment and the being interacting with it (Turvey, 1992; Stoffregen, 2003). For example, for an active young child a sapling tree’s thin, close limbs might afford climbing, but that would not be the case for an infant who is unable to climb or for an adult who might break the branches. Thus affordances can guide and constrain action (Harrington, 2008, unpublished). According to Gibson (1979/1986, p. 143), “the possibilities of an environment and the way of life of an animal go together inseparably.”

One of the issues discussed within the field of ecological psychology is whether an affordance exists as a property of the environment or as a property of the animal-environment system (Turvey, 1992; Stoffregen, 2003). In our view, the theory-driven answer can be different from the pragmatic answer. In theory, an affordance is a property of the animal-environment system. After all, the exact same physical attribute in the environment will often provide an affordance to one type of person but not another. But having said that, we think it is often pragmatically useful to hold a specific category of person as a constant – for example, to hold constant the referent of a child – and then to talk about affordances of the landscape itself, as we did for the sapling tree with thin branches. Both perspectives have merit. We will come back to these ideas later.

### Evolutionary Psychology

Decades ago, Wilson (1984) coined the term biophilia to refer the genetic predisposition that humans have to affiliate with biological life. The mechanism for this nature affiliation is, according to Wilson (1993), that “a certain genotype makes a behavioral response more likely, the response enhances survival and reproductive fitness, the genotype consequently spreads through the population, and the behavioral response grows more frequent (p. 33). In other words, genes that lead to behaviors that enhance survival tend to reproduce themselves (since they are in bodies that procreate more rather than less), and thus these genes and correlative behaviors grow more frequent. For empirical support (see Kahn, 1999, 2011 for summaries), studies have shown that even minimal connection with nature – such as looking at it through a window – can increase productivity and health in the work place, promote healing of patients in hospitals, and reduce the frequency of sickness in prisons. Other studies have shown that when given the option humans choose landscapes that fit patterns laid down deep in human history on the savannas of East Africa. Direct contact with animals has been shown to greatly benefit a wide range of clinical patients: from adults with Alzheimer’s disease to autistic children.

In terms of its theory and empirical support, biophilia has in recent times largely merged with and provided further momentum to the field of evolutionary psychology, which seeks to show that the properties of human social life are the result of evolved adaptations, and thus deeply rooted in our ancestral heritage (Barkow et al., 1992). This theory does not propose that such properties are immutable, or that they are not substantively shaped by culture. But it does mean that to understand the origins and significance of properties of human social life one needs to go back tens of thousands years in our evolutionary history, and sometimes longer.

In this article, as we articulate our model of child–nature interaction patterns, we will be seeking to show that the patterns are ontogenetically and phylogenetically significant. For ontogenesis, we draw on constructivist psychology and ecological psychology to speak about developmental mechanisms, and direct potential outcomes and developmental endpoints that promote human health, mental wellbeing, and human flourishing. For phylogenesis, we draw on evolutionary psychology to show that some of the patterns gain particular meaning because they go far back in our evolutionary heritage, and sustain us still.

## Interaction Patterns

**FIGURE 1:**
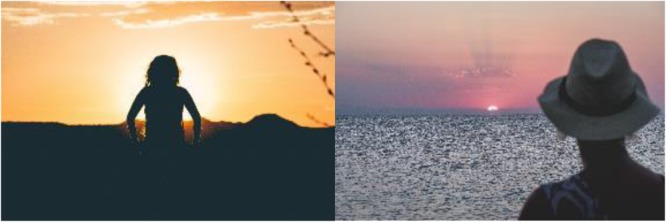
Interaction pattern: *watching the sun set.*

Think about an interaction in nature that you have had that was meaningful. Now characterize it in such a way that you could imagine many such examples of it happening, and even though each example would be at least a little different from the others you would not have a problem recognizing each one as essentially the same form of interaction. If possible, in describing your interaction, include a verb of what you are doing and a noun for the nature that you are doing it with. At that point you probably have an interaction pattern. For example, you have likely enjoyed *watching the sun set* many times in your life. Each time is at least a little different: the weather and colors are never identical; one time you might be on flat land watching the sun set over the hills in the distance, and another time you might be *watching the sun set* over the ocean (**Figure 1**). But no matter the differences, you know it when it’s happening. You can say, “yes, I’m *watching the sun set* now.” That’s the idea of a “pattern” – not in the sense of a cookie-cutter pattern where each form (cookie) is identical, but that of a unified underlying structure of human–nature that can be enacted in an endless number of unique ways. In brief, interaction patterns refer to characterizations of essential features of interaction between humans and nature, specified abstractly enough such that countless different embodied versions of each one can be uniquely realized given different types of nature, people, and purposes. To date, Kahn and his colleagues have generated over 150 human–nature interaction patterns, with photos and descriptions for many of them (Kahn et al., 2010, 2012, 2018a,b; Kahn and Weiss, 2017).

Our pattern work draws on the work of Christopher Alexander and his colleagues (Alexander et al., 1977; Alexander, 1979) who generated 253 patterns in the built environment that they believe engender meaningful human living. According to Alexander, a “pattern describes a problem which occurs over and over again in our environment, and then describes the core of the solution to that problem, in such a way that you can use this solution a million times over, without ever doing it the same way twice” (p. 10). For example, one of their patterns is titled “Light on two sides of every room.” They write: “The importance of this pattern lies partly in the social atmosphere it creates in the room” (Alexander et al., 1977, p. 748). There is now a body of work that has extended Alexander’s idea of patterns into the fields of ubiquitous computing (Chung et al., 2004), software engineering (Gamma et al., 1995; Gabriel, 1996), interaction design (Borchers, 2001), human–computer usability (Graham, 2003), and human–robot interaction (Kahn et al., 2008).

Granted, there are other ways that researchers have used the idea of patterns, often with a more experimental or at least quantitative focus, often involving sequential analysis and observational methods across different populations, including infants, married couples, and non-human primates (Sackett, 1978; Bakeman and Gottman, 1987; Magnusson, 2000). But what we offer here is more along the lines of Alexander in terms of the robust qualitative nature of the patterns. We emphasize this point so as to establish that there are different ways that fields have conceptualized and used the idea of patterns, and explored interaction, and that one way does not preclude another.

### The Continuum of Interaction Patterns: Wild to Domestic

Wildness refers to that which is untamed, unmanaged, not encompassed, self-organizing, and unencumbered and unmediated by technological artifice (Shepard, 1982, 1998; Rolston, 1989; Foreman, 1991; Kahn and Hasbach, 2013a,b). We can love the wild. We can fear it. We are strengthened and nurtured by it (Rolston, 1989; Turner, 1996).

One important feature of interaction patterns is that not only can they be instantiated (enacted) in endlessly unique ways, but those instantiations are themselves usually along the continuum of wild to domestic forms of human–nature interaction. For example, there is the interaction pattern of *movement away from human settlement, and the return*. In Paleolithic times, hunters would leave nomadic campsites and go out in search of animals, and gatherers would leave in search of roots, tubers, nuts, berries, and other plant life. The further out they went, the more they left the safety of the larger group. Both hunters and gatherers would then return, hopefully (but not always) with their bounty, looking forward to the re-union with their group, and greater safety. When a person now hikes for an afternoon into the mountains, it is a less wild form of this interaction pattern; and more domestic still when a person hikes for 20 min out on a park trail.

Granted, wildness is a contested construct. One line of scholarship, for example, has shown how wilderness is largely a cultural construction (Cronon, 1995). From this perspective, it is not the case, for example, that when Europeans began to inhabit North America that they encountered a pristine, untouched wilderness. Rather, the land was an inhabited landscape by Native Americans, and a partly managed landscape at that. Or the Wilderness Act of 1964 in the United States created a legal definition of wilderness, and then partitioned off 9.1 million acres of federal land that was then called “wilderness.” This scholarship has merit; but our position is that there is a good deal of difference between the idea of wilderness and wildness. Wildness, as defined above, has properties – such as an entity or agent being untamed, unmanaged, not encompassed, and self-organizing – that may often be found in designated wilderness areas, but is not synonymous with it. For example, all, a weed growing up through a crack in urban concrete in Tokyo has an aspect of wildness about it that is not embodied in a bonsai plant that may be right next to it. Thus in our view, our focus on wildness (as opposed to wilderness) is not so vulnerable to cultural critiques of this literature.

### Keystone Interaction Patterns

There is no limit to the unique ways that human language can be spoken. It is endless, infinite. That said, much of what we say uses common words, and even phrases: “See you there.” “It’s good to meet you.” “I’ll text you when I leave.” “I’m happy for you.” “I love you.” “What’s the weather tomorrow?” “Let me check.” Some of these phrases can be understood as particularly important not only because they are common but because they play important roles in facilitating human–human interaction. For example, the common phrase “Hello, how are you?” initiates an introduction between two people, and demonstrates an initial (if perfunctory) concern of the person initiating the contact.

Interaction patterns have a somewhat similar structure. On the one hand, there is no limit to the number of interaction patterns that can be characterized. In part, this is because interaction patterns can be characterized into smaller and more discrete forms. For example, there is the interaction pattern of *walking into a body of water*. You can *walk into an ocean, walk into a lake, walk into a river*, and *walk into a swimming pool*. If you’re *walking into the ocean*, you can *walk in over one’s ankles, walk in over one’s knees, walk in over one’s waist, walk in over one’s chest*, or *walk in over one’s head.* For most people, each of these interaction patterns lead to different experiences of the human body in the water. So for most people, it can be useful to use language to make these distinctions. But anybody can say “well, for me, I notice a distinction between getting my toes wet and then wading above my ankles.” Many of us would say, “oh, yes, that makes sense to me, too.” We could call that interaction pattern, *getting toes wet in the ocean.* But at some point the distinctions may get idiosyncratic to a particular person. If someone says “I notice a distinction in going into the ocean just at my knee cap and 1/10th of an inch above my kneecap,” it seems likely that most of us do not experience that distinction as important. But if someone thinks the interaction pattern is important to them, then that’s fine. It is not an issue of right or wrong.

On the other hand, some interaction patterns play more important roles than others, and that can depend on the people involved and their sensibilities, vulnerabilities, and goals; and the nature involved at a specific time. For example, if you are supervising young children who are playing at the seashore, and the waves are breaking at around a height of two feet, you might tell the children “don’t get into the water higher than your knees unless I’m with you!” The key distinction you are making is *getting into ocean at knee-level*, for that is what you believe is the safety spot for these children with their capabilities with these specific waves. Or let us say you are designing a city that seeks to develop an urban region with a lake within it. You might well argue that a particularly important interaction pattern is *walking along the edges of water and land*, or casting it more narrowly for this specific context: *walking around the edge of a lake*. This might well be the critical interaction pattern that you need to design for, which means keeping houses and development away from the edge of the lake itself, and creating a pathway around the lake, so that people can *walk around the lake*. Knowing that this interaction pattern is a particularly important one helps to provide the language for urban design and urban sustainability (Hartig and Kahn, 2016; Kahn et al., 2018b). Such interaction patterns have been referred to as *keystone* interaction patterns.

A keystone interaction pattern is any interaction pattern that plays a disproportionately large role in human–nature interaction because (a) it occurs frequently, (b) it is itself hugely beneficial or meaningful, (c) it engenders dozens or even hundreds of complementary, subsidiary, or overlapping interaction patterns, and/or (d) its loss leads to the subsequent loss of dozens or even hundreds of complementary, subsidiary, or overlapping interaction patterns (cf. Kahn et al., 2018b). This use of the term *keystone* partly mimics the term *keystone species* in conservation biology, which refers to a species (such as a top predator) that has a disproportionate benefit to its environment relative to its abundance (Mills et al., 1993; Paine, 1995). For example, if the wolf (a keystone species) is removed from such areas as Yellowstone National Park, then elk grow more abundant and stationary, overgrazing vegetation, which leads to the loss of habit, increased erosion, and the loss of biodiversity (Eisenberg, 2013).

On occasion, it is possible that several interaction patterns are themselves combined and then elevated to the level of a keystone interaction pattern, or that some other aspect of the interaction is important enough to append to the core verb/noun structure of a keystone interaction pattern. An analogy can be made to the naming of different types of soup. Some soups are named by their main ingredient, or at least for their distinctive taste. There is, for example, potato soup, lentil soup, chicken soup, carrot soup, leek soup, split pea soup, and miso soup. But sometimes a soup is named based on two of its ingredients. For example, chicken noodle soup usually has many ingredients other than chicken and noodles (e.g., carrots, celery, onion, garlic, and olive oil), but it is not called “chicken celery soup” or “chicken garlic soup” but chicken noodle soup because the chicken and noodles structure its main flavor and the experience of eating the soup. Something similar can occur with interaction patterns. This point will become clear when we discuss the interaction pattern *lying on earth in solitude.*

### A Nature Language for Interaction Patterns

Interaction patterns can be thought of as a little bit like words in a dictionary. Words can be defined as individual entities, but they exist in our lives mostly in relation to one another, just as you are reading the words on this page. Similarly, interaction patterns can be defined as individual entities, but are experienced with many other interaction patterns in often overlapping and sequential ways with semantic coherence. For example, you can be *walking a trail* and *stepping over a log* while *seeking to get to a desired bluff top spot* so that you can then *look out to the snow-capped mountains*, and while you do so you might be enjoying the *sun shining on your skin, feeling a light wind*, and decide to *pick some wild blueberries* and then while you’re *kneeling on the ground* you might catch a quick look and *watch a Cooper’s hawk chase down a quail*, as you *swat a mosquito that has landed on your arm.* Human interaction with nature is endlessly varied, endlessly deep. In such ways, interactions can be combined in human discourse, and help form a *nature language* — a way of speaking about patterns of interactions between humans and nature, their wide range of instantiations, and the deeply meaningful and often joyful feelings that they engender (Kahn et al., 2012).

But there is another and perhaps more substantive way that a nature language can be used. It is to use language to speak about any specific interaction pattern, especially about a keystone interaction pattern, in order to help others understand how the interaction can be enacted, and what it feels like to experience.

This is important because in current times the natural world is getting destroyed quickly, across generations; and as we lose nature, we lose the knowledge of how to interact with nature and how wonderful it feels to do so; in turn, that loss leads to our loss of language of how to speak about interacting with nature. The anthropologist Davis (2007) writes that when indigenous cultures lose their language their indigenous way of life dies; that language needs to be spoken, it needs to be “lived” in order for a culture to survive. Similarly, we need to help revive a diverse and deep *nature language* if we are to help reverse the course of environmental destruction. Thus it is important not only to characterize interaction patterns, but to provide a rich narrative for many of them, especially keystone interaction patterns. Such narratives can draw from personal experiences, accounts of indigenous peoples, the historical record, nature writing, evolutionary psychology, and empirical studies. Each narrative helps to make interaction patterns “alive” through words so as to help others know what is possible. For this reason, when we – in the latter part of this article – characterize 7 keystone interaction patterns in a nature preschool, we will be using a nature language to help articulate the patterns themselves.

### Modeling Child–Nature Interaction Through Interaction Patterns

In its basic sense, a model is a simplified description of the information of a phenomenon in the world with the objective of making the phenomenon understandable. There are many types of models, including “probing models, phenomenological models, computational models, developmental models, explanatory models…theoretical models, scale models, heuristic models, caricature models, didactic models…mathematical models…formal models, analog models and instrumental models” (Frigg and Hartmann, 2012).

In our work here, we are seeking to model human–nature interaction through the heuristic of interaction patterns. Interaction patterns represent a simplification of the world with the goal to make the world more understandable. Our model incorporates attributes of what is called a phenomenological model insofar as it seeks to represent observable properties of their targets, while incorporating principles and laws associated with scientific theories (Frigg and Hartmann, 2012). In our case, our model incorporates principles, as discussed earlier, from theories of constructivist psychology, ecological psychology, and evolutionary psychology.

Validation of the model occurs in several complementary ways. Given its phenomenological stance, there is face validity based on one’s own direct experience. One can ask, “does this interaction pattern make sense to me based on my own experience in nature?” If it doesn’t, then while it could still be an interaction pattern, it may not be an important interaction pattern. Then there is the question: “Is the interaction pattern within the realm of human possibility?” For example, there is the possible interaction pattern which has an appropriate linguistic structure of a relevant verb and nature noun, *stepping over an ocean*, which is just not physically possible. Thus *stepping over an ocean* is not a valid interaction pattern. Validation of interaction patterns is further established the more parsimoniously they correspond with the theories of constructivist psychology, ecological psychology, and evolutionary psychology. In addition, the idea of keystone interaction patterns involves an empirical claim that they occur frequently in a specified population and/or are particularly important and meaningful to that population, and/or engender dozens or even hundreds of complementary, subsidiary, or overlapping interaction patterns, and/or if lost leads to the subsequent loss of dozens or even hundreds of complementary, subsidiary, or overlapping interaction patterns. These are empirical claims that can be used to test the validity of a keystone interaction pattern. Finally, part of the validity of our model lies in whether it can lead to testable hypotheses – not for the model itself (as in a climate change model), but in terms of leading to predictions of the world using the interaction patterns.

In what follows, we seek to provide a proof of concept that human–nature interaction can be modeled using the heuristic of interaction patterns, supported with a nature language. This is a beginning venture, not an end. We focus on what seem to us keystone interaction patterns, and begin to validate our model based on the above epistemological criteria.

## The Proof of Concept

Over a period of 7 months, we have been filming children interacting with nature in a nature preschool in Seattle, WA, United States. The school is Fiddleheads Forest School, at the University of Washington Botanic Gardens, directed by one of us (Harrington). The children (ages 3–5 years old) and teachers spend all of their time outside, in a matrix of trees, in one of two classrooms located in the University of Washington Botanic Gardens. These botanic gardens are open to the public in this fast-growing city. We divided each of the two outdoor areas into five different filming zones, and through a randomized time-sampling methodology are filming children. Our analysis is based almost entirely on the observed digital video footage; though our videos did capture diffuse sound, and so occasionally we had faint child language to work with, too, in interpreting the behavior. One of the strengths of this method is that it is minimally intrusive in the children’s interactions with nature, and is highly systematic in randomly covering all of the landscape. One of the limitations is that we did not interview the children about how they themselves understood their interactions (Turiel, 1983; Kahn, 1999), which itself could become a future study.

As a step forward in characterizing child–nature interaction in a nature preschool, we offer here seven keystone interaction patterns that have been emerging from our data.

This study was carried out in accordance with and with the approval of the Institutional Review Board at the University of Washington, the Human Subjects Division. The parents of all the child participants gave written informed consent in accordance with the Declaration of Helsinki. For those parents who did not give permission for their child to participate, we then explicitly kept them out of all of our video footage, either by zooming in on only the children for whom we had permission (in our time-sampling methodology), or if that was not possible given their close proximity to other children we were filming, we stopped filming all of the children for that duration. Occasionally a parent gave permission for their child to participate but if the child’s image was to be used in a publication, such as this one, they requested the child’s face be blurred, which we did.

### Using One’s Body Vigorously in Nature

In the United States, about 75% of children ages 5–10 do not get enough exercise (Hendrick, 2011), which increases to over 90% for adolescents (Li et al., 2016). Exercise strengthens the heart, lungs, and bones, decreases the likelihood of developing obesity, decreases the risk factors for diseases like type 2 diabetes and heart disease, can reduce anxiety and depression, and promotes positive mental health (Youth Physical Activity, 2009).

From an evolutionary perspective, such outcomes are not at all surprising (Wilson, 1984; Kellert and Wilson, 1993; Kahn, 1999, 2011). For tens and hundreds of thousands of years, we as a species came of age using our bodies vigorously in nature. That was a requirement for survival. For example, in her ethnography based on living with Ju/Wa bushmen in the Kalahari desert in the early 1950’s – at a time when their way of life may well have embodied much of the hunter-gatherer life from 50,000 years earlier – Thomas (2006) documents that the Ju/Wa women she went foraging with would sometimes carry home 50–80 pounds of tubers, roots, and nuts. The Ju/Wa women themselves weighed about ninety pounds. Thomas estimates that these women walked about 1,500 miles a year. Hunting, too, was physically strenuous. One method of hunting a bull eland was especially demanding. The mature bull eland, in particular, with large amounts of body mass, could be overcome on especially scorching days by a runner who kept at him mile after mile. The runner could not match the eland for speed, for the eland sprints at 35 miles an hour. But eventually, after many hours of being chased in the heat, the eland overheats and can run no more. “Then the hunter, with the last of his strength, can catch up and grab him by the tail, then kill him with a spear if he brought one, or he can push the eland over and lie on his neck to keep him from struggling and clamp his hands over the eland’s nose and mouth to stop his laboring breath” (Thomas, 2006, p. 32). Thus from this evolutionary perspective, our bodies and minds are optimally programmed to thrive through using our bodies vigorously in nature, which as modern people we have now reduced to what we call “exercise.”

**FIGURE 2:**
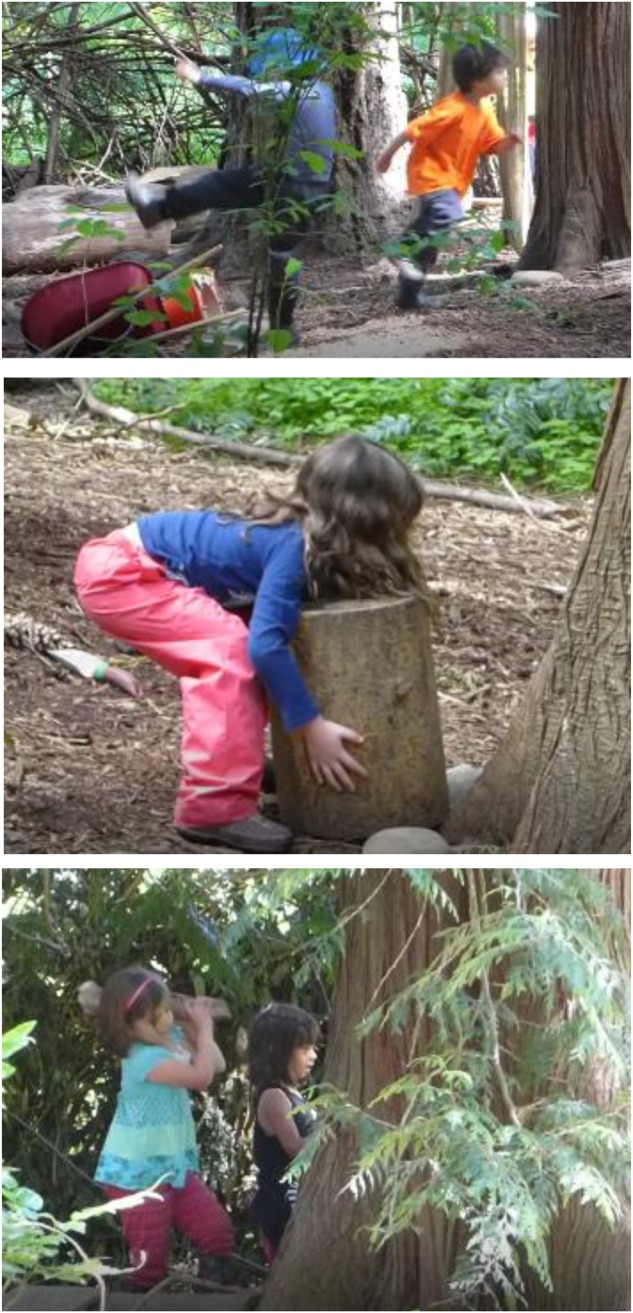
Interaction pattern: *using one’s body vigorously in nature.* Signed informed consent was obtained from the parents of all the children as shown in the photos in this manuscript.

Because *using one’s body vigorously in nature* can be instantiated in so many different ways, and plays such a critical role in human health and wellbeing, it is a good keystone interaction pattern to start with here. The ways this interaction pattern is enacted in Fiddleheads are not so surprising. For example, **Figure 2** shows children *running on uneven ground, kicking at a wheelbarrow, lifting a stump*, and *hitting a tree with a heavy branch*.

Our point here is not to describe all of the ways that these children use their bodies vigorously in their nature preschool (though that is a worthwhile future goal), but to provide the keystone interaction pattern by which anyone can then begin to characterize subsidiary interaction patterns whenever they see it occur with children. For example, if you see children *dancing on the ground* or *swinging on a tree limb, tumbling down a hill, running up a hill, chasing butterflies*, or *moving heavy rocks* – you can say “ha, within a very broad framing of child–nature interaction, there’s something common and important to all of it: they are *using their bodies vigorously in nature*.

With this keystone interaction pattern in hand, one can then generate important hypotheses. For example, it is possible that children in a nature preschool are more “active” than children in a traditional preschool with inside classrooms. However, such differences in children’s activity may or may not show up if it is measured by a pedometer for steps walked. Rather, a more specific hypothesis is that children in a nature preschool use their bodies more vigorously than in a traditional preschool as measured by the total number and duration of engagement of the subsidiary interactions patterns of this keystone interaction pattern.

### Striking Wood on Wood

The previous keystone interaction pattern – *using one’s bodies vigorously in nature* – is framed at a very broad level in the sense that it hierarchically encompasses at least hundreds of more specific forms of interactions that themselves constitute interaction patterns. We mentioned a few above: *running up a hill, chasing butterflies, and moving heavy rocks*. Given a specific population, landscape, situation one is trying to model, and audience that one is speaking to, a subset of a keystone interaction pattern can also constitute a keystone interaction pattern. We think that is the case for *striking wood on wood.* It is shown being enacted at the bottom of **Figure 2** where a girl with a club-like piece of wood is in the process of using it to strike the trunk of the large tree.

**FIGURE 3:**
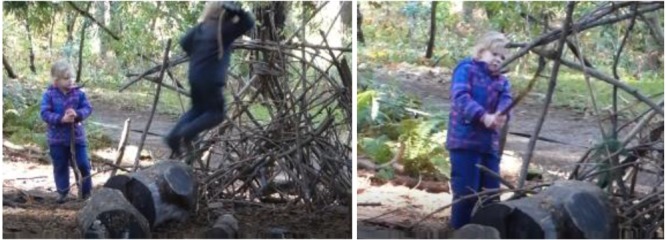
Interaction pattern: *striking wood on wood.*

As a more complex instantiation of this interaction pattern, consider an event we observed where a boy (**Figure 3**) jumps off of a stump and with a stick in hand slams his stick repeatedly into the wood structure. In turn, a girl (also in **Figure 3**) initially watches him, and then decides to try out something similar. She picks up a stick and starts striking it, with increasing vigor, against other wood. If you were watching this video data, you would see how the initial affordance of striking wood on wood appears to lead the girl to new perceptions of affordances, such that she understands that she can strike harder. Our impression is that she is not so much trying to destroy something but trying to figure out the properties of *striking wood on wood*: how to strike, and how increased force leads to different outcomes. Sometimes when children enacted this interaction pattern, they struck with a light branch, heavy branch, or club-like branch. Sometimes they extended this interaction pattern beyond wood on wood, and struck with a tool, such as with a garden hoe or shovel. Sometimes what was being struck was a live tree, live branches, logs, stumps, or the ground.

Why do children enact this interaction pattern in play? Perhaps it is because that this form of interaction lies deep within our evolutionary history. It is primal. It is the woman gatherer 50,000 years ago, kneeling on the African desert sands, using a hefty digging stick to dig 10 or even 18 inches deep for tubers, striking the earth again and again (Thomas, 2006) for survival. It is the hunter striking an animal’s body with a spear, seeking to pierce the animal’s heart (Marshall, 2002). It is the woodsman chopping wood, and striking the wood round again and again, to split it, so as to have the pieces by which to build and sustain a fire. What we are likely seeing enacted here, then, are the ontogenetic origins from our phylogenetic past.

### Constructing Shelter

This interaction pattern also goes far back in our evolutionary heritage. As humans, we have constructed shelter for perhaps as long as we have been a species. During Paleolithic times, the constructions would have modified natural affordances of the landscapes, such as caves; or used materials in hand, and led, for example, to light thatched huts on the savannahs of Africa, easily constructed and easily left behind in nomadic hunter-gather life (Thomas, 2006).

**FIGURE 4:**
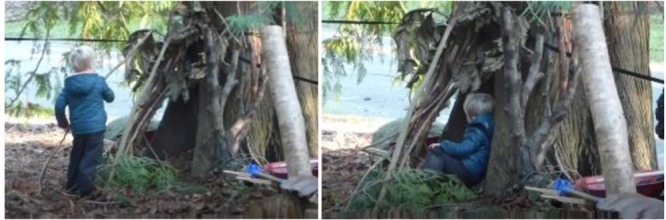
Interaction pattern: *constructing shelter.*

This form of children’s interaction overlaps with what is referred to in the literature as place-making (Sobel, 1993; Nabhan and Trimble, 1994; Chawla, 2002; Sampson, 2012, 2015). For example, in **Figure 4**, the child is modifying the hollow of a tree by leaning branches up outside the hollow, thereby creating a little more protection and privacy. Then he engages in another interaction pattern: *leaning against tree*, and thereby finds respite.

### Being in Solitude in Nature

In Milton’s (1674/1978) epic poem *Paradise Lost*, he writes “For solitude sometimes is best society, /And short retirement urges sweet return” (book IX). He was writing of Adam going off alone for a while in the Garden of Eden. Others emphasize that *being in solitude in nature* leads to deep experience. Muir (1976, p. 314) put it this way: “Only by going alone in silence, without baggage, can one truly get into the heart of the wilderness. All other travel is mere dust and hotels and baggage and chatter.” Of course solitude is relative to person and place. For Muir it might mean hiking a week in the Sierras without meeting another person; for a child it might mean spending a few minutes alone next to a favorite tree.

**FIGURE 5:**
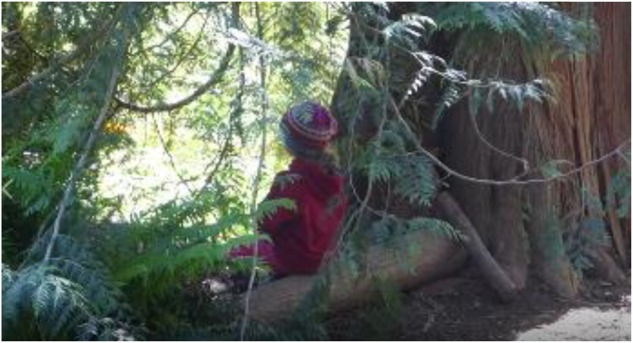
Interaction pattern: *being in solitude in nature.*

The girl in **Figure 5** had just been involved in an altercation, and went into this enclosed tree area. One of us (Weiss) was already filming in this general area and continued filming. The girl noticed the researcher, and could not seem to find the peace she needed. She fidgeted, for example, with various sticks and leaves in her hand. After several minutes, she got up and approached the researcher and politely asked if the filming could stop so as to give her some alone time. She then went back to her spot and sat by the large tree. The researcher stopped filming and moved a little ways away. Children at Fiddleheads often take advantage of the privacy that more wild or secluded parts of the landscape affords. As in this instance, it sometimes appears to be an effective mechanism for self-regulating and regrouping: processing something difficult that had just occurred with other people. Other times the solitude seems to afford some of what Muir was writing about: it allows children to get more into the heart of nature itself, as when a girl at Fiddleheads went alone into a more wild part of the landscape and stood still for a bit and then started to enact the interaction pattern *calling the birds*.

### Lying on Earth

Modern people are losing direct physical contact with the earth. That loss is likely due to ignorance and happenstance. Ignorance in that we have forgotten how good it feels to have one’s body in contact with the earth. Happenstance in that the urban world is increasingly paved such that there is little ability to lie on earth; and even when there is opportunity, we often design nature areas to prevent this interaction pattern under the guise of comfort. For example, it is likely that many times you have arrived at a beautiful resting spot in a park or nature preserve, perhaps alongside a creek, or on a bluff top overlooking a beautiful view, and there is a bench there for you to sit on. So you sit on it. That is an affordance of the bench. But in doing so you have passed over the affordance of the earth itself. It is an enjoyable feeling to place one’s body in contact with the earth. You feel its contours, its heat or cold. Perhaps you place your hands in the earth. Or on a hot summer’s day, perhaps you take your shoes off and place your feet into the soil. There is emerging scientific literature that shows the cognitive and health benefits of skin in contact with soil. For example, a strain of bacterium found in soil, mycobacterium vaccae, appears to improve cognitive functioning, and triggers the release of serotonin, which in turn elevates mood and reduces anxiety (Lowry et al., 2007). There is also emerging thought that contact with the ground helps to balance the body’s electrobiochemical system (Adams, 2012). For example, Chevalier (2014) writes:

The body is a highly intelligent electrobiochemical system that is strongly influenced by its internal electrical environment. Countless electrical charges within this system regulate countless biochemical reactions, including enzymatic transformations, protein formation, and pH (acid/alkaline) balance. In this complex arrangement, the Earth’s surface electric potential serves as the body’s stabilizing reference point or ground…[direct] contact with the surface of the Earth maintains the body’s electrical stability and normal functioning of its self-regulating and self-healing mechanisms. (p. 255)

**FIGURE 6:**
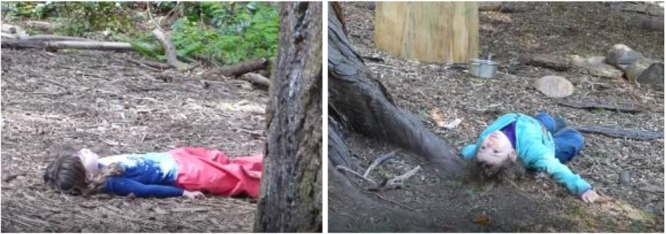
Interaction pattern: *lying on earth.*

Both girls in **Figure 6** are enacting this interaction pattern of lying on earth. In addition, the girl in the first photo is enacting what could be called a combinatorial keystone interaction pattern insofar as she is also *being in solitude in nature*. Specifically, this girl had just had an altercation with another child and, in our interpretation, she wanted to regroup. She walked to this area and seemed a little agitated, and then wandered around a little, almost as if she was trying to find the right spot where she felt most comfortable to lie down. And then she did. And then it is as if the earth helped ground her emotionally. This form of interaction – *lying on ground in solitude* – could itself be elevated to a keystone pattern because it seems to us especially powerful as a form of communion with nature.

### Cohabiting With a Wild Animal

It has been said that one of the overarching problems of the world today is that we see ourselves as dominating over nature, rather than cohabitating, coexisting, and affiliating with it (Kahn, 2009, 2011; Kahn and Hasbach, 2013a,b). Perhaps the basis for cohabitating grew out of necessity in Paleolithic times. For example, Thomas (2006) recounts an experience one evening when she was living with the Bushmen of the Kalahari desert in the early 1950’s when four lions walked into the Bushmen camp. The Bushmen had no way of killing a lion. One of the men took a flaming branch from the fire and in a firm tone, without ever taking his eyes off the lions, talked to the lions, and then ended by telling the lions respectfully but firmly that they could not be here, and to go! The lions watched the person, and “then gracefully they turned and vanished into the night” (p. 151). Of course, for the most part the lions were not interested in eating or harming people; lions and humans coevolved together for hundreds of thousands of years in that landscape. There was enough space for both of their species to live and to thrive. As our species then evolved, and we gained the ability to control, use, and destroy more and more of our environment, and created the technological tools to do so faster, and populated faster, our wellbeing if not our very existence on the planet now comes under threat. One solution is to rediscover how to cohabit with the wild (Kahn and Hasbach, 2013a,b). It becomes a necessity again, no longer because of our limited ability to control nature but because of our seeming inability to control ourselves.

A subset of the interaction pattern *cohabiting with the wild* that we think speaks powerfully to what occurs at Fiddleheads Forest School is *cohabitating with a wild animal*. They are not wild animals like lions, obviously. But the animals such as birds, spiders, and worms are wild insofar they are autonomous, self-regulating, and not depending on the care of humans to live.

**FIGURE 7:**
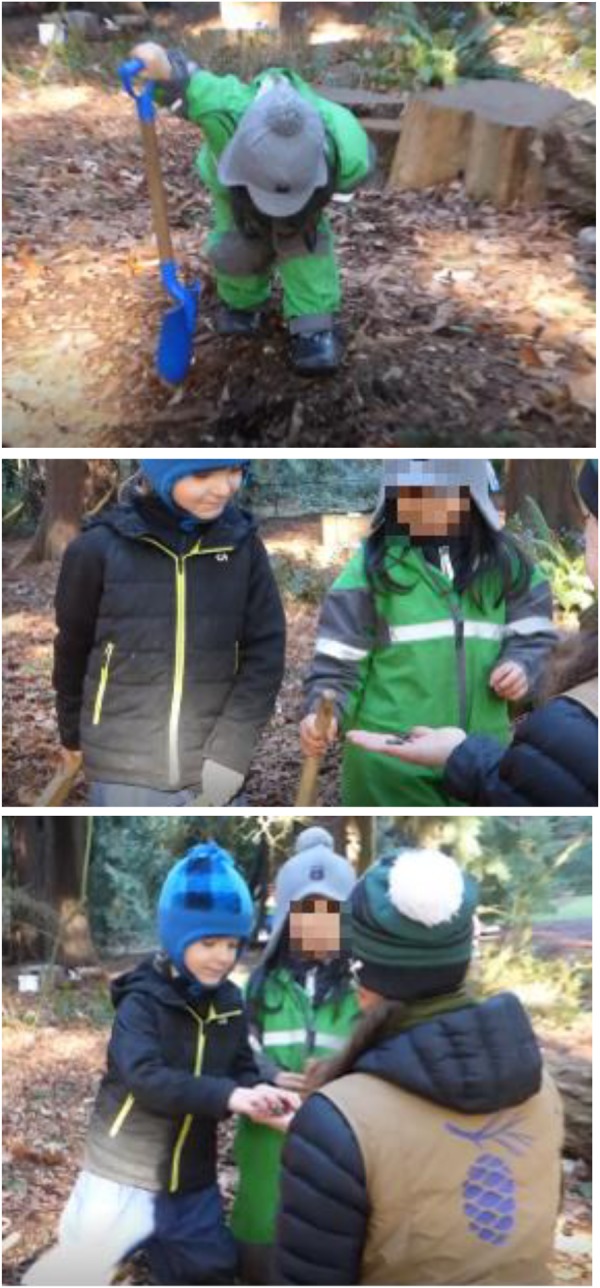
Interaction pattern: *cohabiting with a wild animal.*

Here is an illustrative case in point of this interaction pattern. A few children were digging and moving earth in a wheelbarrow when the girl in **Figure 7** noticed a worm in the soil. The boy had come close to running it over as he was moving quickly with the wheelbarrow in hand. The girl initially displayed aversion to the worm, but that soon changed to fascination. A teacher noticed what was going on and then kneeled down and picked up the worm, placed it in her palm, and showed it first to the girl, who began to get comfortable being in the presence of the worm. The boy saw what was happening and, in our interpretation, wanted to get on with his construction without harming the worm, so he walked over and without hesitation took the worm out of the teacher’s hand and placed the worm some feet away, out of the construction area. He then got back to work. Both children were cohabitating with this animal: the girl by being in its company, and learning to appreciate and take delight in it; the boy by being able to find a way to continue with his life while allowing the animal the space to continue with its life. Notice, too, how the teacher was able to foster this interaction by bringing the animal to the attention of the children, demonstrating that the animal was not harmful (by having it in the palm of her hand), and then giving children the space to figure out what would happen next.

### Being Outside in Weather

Some interaction patterns are so pervasive that they can escape notice, even if they are important for humans to enact. *Breathing air* is an example. We hardly think of it as a form of interaction – that is, until it becomes hard to breathe. Then we might emphasize the adjective *clean* as in *breathing clean air*. There are cities in this world where *breathing polluted air* has equivalent health impacts of inhaling 2–5 dozen packs of cigarettes a day.

**FIGURE 8:**
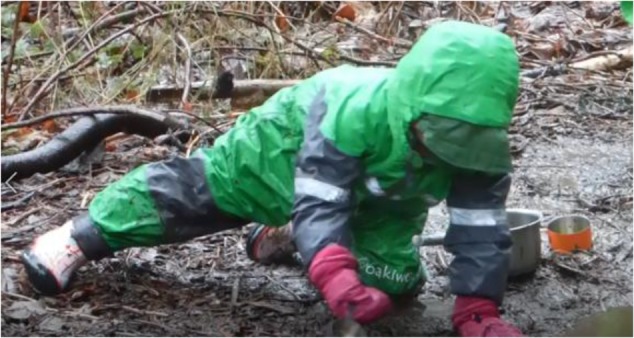
Interaction pattern: *being outside in weather.*

One such pervasive interaction pattern, and a defining characteristic of nature schools in general, is *being outside in weather.* The children at Fiddleheads have no inside space. It rains a lot in Seattle. Occasionally it sleets or even snows. Sometimes in late Spring the sun shines and it is hot. Children spend all of their time *being outside in weather*. **Figure 8** shows a child enacting this interaction pattern on a January day of heavy rain. She is well dressed!

*Being outside in weather* is not only pervasive, but helps make possible many of the interactions that occur in a nature preschool. This is worth naming, and keeping in mind, when discussions occur about whether it is important to balance outside time with inside time in any specific nature school. It is also the case that this interaction pattern helps connect children with perhaps the wildest parts of nature that they have access to if the school is in an urban environment. For weather by definition is wild insofar as you do not control it: it is self-organizing, and it is big nature, some of the biggest, and while it can be nurturing and healing, it can also be fierce, and if you are not careful it can kill you. In this sense, children learn to respect nature, and to cohabit with the wild.

## Additional Keystone Interaction Patterns

We have characterized seven keystone interaction patterns that have emerged from our observing children at Fiddleheads Forest School, and provided a nature language about them: *using one’s body vigorously in nature, striking wood on wood, constructing shelter, being in solitude in nature, lying on earth, cohabiting with a wild animal*, and *being outside in weather*. In two other recent venues, we have characterized an additional 14 keystone interaction patterns (Kahn and Weiss, 2017; Kahn et al., 2018b). Thus here, in **Table 1**, we bring together all of the keystone interaction patterns to date, and describe them briefly, and note the ontogenetic and/or phylogenetic significance of each of them. For the reader interested in a fuller explication of any of the additional interaction patterns, we delineate which additional pattern is discussed in which venue. **Table 1** may be especially useful for practitioners insofar as it provides a condensed description of what children are actually doing at this nature preschool, and potentially other nature preschools with a similar landscape. For example, if a director of a nature preschool is trying to explain to parents what their children are actually doing each day outside, and why it is probably important for them, the director could draw directly on whatever parts of this table seem most relevant.

**Table 1:** Toward modeling child–nature interaction in a nature preschool: 20 keystone interaction patterns.

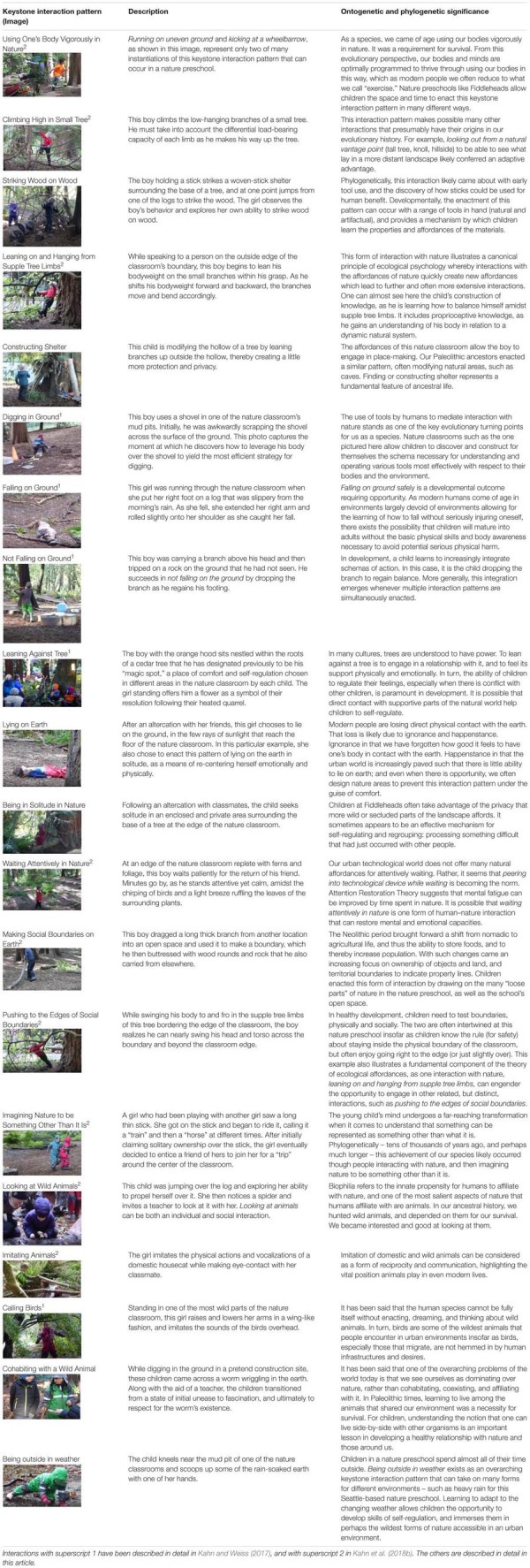

## Discussion

The qualitative research presented in this article seeks to make a compelling case – as a proof of concept – that child–nature interaction can be modeled in a nature preschool based on interaction patterns.

In support of our proof of concept, we provided a summary of the model’s underlying theory which draws from constructivist psychology, ecological psychology, and evolutionary psychology. We also provided an account of interaction patterns, and discussed the phenomenological model we are developing, and the issue of validation. Then we moved to the substance of our research. We provided a description of seven keystone interaction patterns that have emerged from our observational data, along with an extended *nature language* to convey their ontogenetic and phylogenetic significance. Finally, we integrated these 7 interaction patterns with 13 other patterns published elsewhere. Thus, for the first time, we have in this article presented and synthesized 20 keystone interaction patterns for this nature preschool.

These 20 keystone interaction patterns do not complete the model for two reasons. First, additional qualitative analyses of more keystone interaction patterns is needed, along with a quantitative coding (with assessments of intercoder reliability) of all keystone interaction patterns so as to establish their relative frequency. We think we have most of the keystone interaction patterns identified, but not all of them. That work is currently ongoing, and will be reported at a later date. Second, our model is constructed to be open-ended, and thus is responsive to whomever wants to modify it based on their own sensibilities and goals. For example, it is possible to drill down with greater and greater specificity to name very specific interaction patterns, such as *sitting on log with left foot in the air; sitting on log with left foot extended on ground.* There are thousands of interaction patterns of this sort, if not more. Are they interesting? At this moment, not to us with the lenses that we bring forward, including that of developmental psychology, education, environmental education, and parenting. But if we were specialists in something like “child-sitting ergonomics,” then such interaction patterns could be of particular interest, and be elevated to a keystone level. Assuming that the modeling of the ergonomic child–nature interaction patterns are being done well, then it is not an issue of the modified model being right or wrong, but relevant or not relevant to a particular audience. That is an extreme example, of course. But it is possible that people even with a shared orientation may want to emphasize certain interaction patterns over others; and that is fine, and they would then provide the *nature language* to help others understand why that pattern is particularly significant. Thus in our view, our modeling is both product and process. Both are intellectual contributions.

Part of the strength of our approach to modeling is that it can account for forms of interaction that cut across many if not all nature preschools, while allowing for differences in the way that the interactions are instantiated, or in terms of the subsets of interaction patterns that are called forward to comprise the larger pattern. For example, *using one’s body vigorously* may occur in all nature preschools, but in some schools that may involve *running up a hill* while also *running on flat land* while in other schools, like in the Fiddleheads main classrooms, there are no hills to speak of, so you would not see *running up a hill*. Or *climbing high in small tree* can occur in many different regions with many different species of small trees; though it will not occur in nature preschools where there are no trees. Thus future studies could employ our approach to modeling to compare nature preschools in different geographical locations (e.g., with more or less wild landscapes), or to compare nature preschools to traditional inside preschools in the same location. Along the same lines, our modeling, based on interaction patterns, can account for what is universal and particularistic based on culture and subgroups. Thus future studies could also employ our approach in comparative studies of nature preschools in different cultural settings.

With a working model in hand, one can also move beyond comparative studies and generate and test important hypotheses. For example, one hypothesis that we are currently exploring is whether more relational forms of interaction with nature (such as *calling birds, leaning against tree*, and *imitating animals*) occur in landscapes that have affordances that are more wild. If such a hypothesis bears out, it would speak to the importance, even in modern times, for children to connect to more wild forms of nature to develop relational ways of engagement.

## Data Availability Statement

The full video segments supporting each of the seven keystone interaction patterns will be made available by the authors, without undue reservation, to any qualified researcher.

## Author Contributions

PK: conceived study, co-responsible for leading intellectual work, did most of the actual writing. TW: responsible for data collection, co-responsible for leading intellectual work, assisted with writing. KH: involved with implementation, intellectual work, and writing.

## Conflict of Interest Statement

KH is the Director of the Fiddleheads Forest School where this research was carried out. The other authors declare that the research was conducted in the absence of any commercial or financial relationships that could be construed as a potential conflict of interest.
